# Thyroid Nodules with Indeterminate FNAC According to the Italian Classification System: Prevalence, Rate of Operation, and Impact on Risk of Malignancy. An Updated Systematic Review and Meta-analysis

**DOI:** 10.1007/s12022-022-09729-x

**Published:** 2022-08-31

**Authors:** Pierpaolo Trimboli, Giulia Ferrarazzo, Carlo Cappelli, Arnoldo Piccardo, Marco Castellana, Jessica Barizzi

**Affiliations:** 1 Servizio Di Endocrinologia E Diabetologia, Ente Ospedaliero Cantonale (EOC), Bellinzona, Switzerland; 2 Facoltà Di Scienze Biomediche, Università Della Svizzera Italiana (USI), Lugano, Switzerland; 3 Medicina Nucleare, Ospedale Villa Scassi Hospital, Genoa, Italy; 4 Department of Clinical and Experimental Sciences, SSD Medicina Ad Indirizzo Endocrino-Metabolico, University of Brescia, ASST Spedali Civili Di Brescia, Brescia, Italy; 5 Struttura Complessa Di Medicina Nucleare, E.O. Ospedali Galliera, Genoa, Italy; 6 Ambulatorio Di Endocrinologia E Diabetologia, Poliambulatorio Di Monopoli, Azienda Sanitaria Locale Bari, Monopoli, Italy; 7 Servizio Di Citopatologia, Istituto Cantonale Di Patologia, Locarno, Switzerland

**Keywords:** Fine-needle aspiration (FNAC), Indeterminate nodules, Thyroid, Carcinoma, Risk of malignancy, Italian Consensus for the Classification and Reporting of Thyroid Cytology

## Abstract

A thyroid nodule classified as indeterminate on fine-needle aspiration cytology (FNAC), hereafter referred to as an indeterminate thyroid nodule (ITN), represents a clinical dilemma. The Italian Consensus for the Classification and Reporting of Thyroid Cytology (ICCRTC) divides ITNs into low- and high-risk categories (i.e., TIR3A and TIR3B, respectively) to better manage patients. This study aimed to achieve high-evidence estimates of the prevalence, rate of operation, and risk of malignancy of ITNs, including TIR3A and TIR3B ITNs. This systematic review was conducted according to MOOSE to retrieve all original studies citing ICCRTC. The last search was performed in February 2022. The risk of bias of the included studies was assessed. Separate proportion meta-analyses were performed with a random-effect model using OpenMeta[Analyst]. The online search processed 271 studies, and 33 were finally considered. First, the cancer prevalence among ITNs was 32.4%. Second, the cancer prevalence among TIR3As was 12.4%, with heterogeneity (I_2_ 90%) explained by a linear correlation between sample size and cancer rate (*p* = 0.009). Third, the cancer prevalence among TIR3Bs was 44.4%, with heterogeneity (*I*_2_ 75%) explained by the inverse correlation between sample size and cancer rate (*p* = 0.031). Fourth, the prevalence of ITNs, TIR3A, and TIR3B among FNACs was 29.6%, 12.6%, and 12.9%, respectively, with sample size and TIR3B prevalence being inversely correlated (*p* = 0.04). Fifth, the operation rates of ITNs, TIR3A, and TIR3B were 54.3%, 48.3%, and 75.2%, respectively, and the sample size and TIR3A operation rate were inversely correlated (p = 0.010). These data strongly support the division of ITNs into low- and high-risk subcategories. Importantly for clinical practice, the cancer rate among ITNs is significantly influenced by the study sample size.

**Supplementary Information:**

The online version contains supplementary material available at 10.1007/s12022-022-09729-x.

## Introduction

Thyroid nodules classified as indeterminate on fine-needle aspiration cytology (FNAC), hereafter referred to as indeterminate thyroid nodules (ITNs), represent one of the most relevant clinical dilemmas in the field of clinical thyroidology. Thyroid nodule is a largely diffuse pathological entity that is often incidentally discovered during neck imaging performed following nonthyroidal indications [1]. According to international guidelines [2, 3], in patients with newly discovered thyroid nodule(s), the indication for further diagnostic procedures should be considered. In this context, ultrasound (US)-guided fine-needle aspiration cytology (FNAC) is recognized as the most reliable tool [2, 3]. In fact, FNAC is able to discriminate malignant from benign thyroid nodules with high accuracy. However, a nonnegligible number of FNACs are classified as ITNs, namely a kind of nodule in which a full diagnosis can be achieved only by histological evaluation after surgery. Since the prevalence of ITNs among FNACs is expected to be 20 to 25% and considering that approximately one in three ITNs is expected to be cancer [4], international guidelines recommend managing these patients according to specific clinical context and US features with the aim of avoiding surgeries as much as possible. Then, the indeterminate category is usually divided into two subcategories, such as Thy 3a (ITN with atypia) and Thy 3f (ITN with follicular pattern) in the UK Royal College of Pathologists (RCPath) guidelines [5], AUS/FLUS (atypia of undetermined significance/follicular lesion of undetermined significance), and FN/SFN (follicular neoplasm/suspicious for a follicular neoplasm) in The Bethesda System for Reporting Thyroid Cytopathology (TBSRTC) [6], and TIR3A and TIR3B in Italian consensus for the classification and reporting of thyroid cytology (ICCRTC) [7]. Some systematic reviews with meta-analyses have been published about the rate of malignancy of these subcategories, and they found a cancer rate of 25% (95% CI 20 to 31) for Thy 3a and 31% (95% CI 24 to 39) Thy 3f [8] in UK RCPath, 30.5% (95% CI 24.2–37.0) [9] or 27% (95% CI 23 to 31) [10] in AUS/FLUS, 28.9% (95% CI 26.2–31.6) [9] or 31% (95% CI 28 to 36) [10] in FN/SFN of TBSRTC, 17% (95% CI 12 to 22) in TIR3A, and 47% (95% CI 40 to 55) in TIR3B [11] of ICCRTC. While from a clinical standpoint these results seem to help to accurately guide the management of ITN patients, we must consider that they were obtained only from a series of patients managed and operated in each single institution according to institution-specific clinical guidelines, international or national guidelines, and other factors, such as endocrinologists’ and surgeons’ expertise and patient preference. Then, we must ask ourselves how this selection bias could influence the findings forming the international guidelines. In addition, we have to take into account that ITNs can include highly aggressive follicular carcinoma, which is difficult to identify on US [12] and is not detectable in cytological samples [4].

Following the above critical issues, the present systematic review was undertaken to achieve more robust information about the risk of malignancy among ITNs. Theoretically, to assess the true cancer prevalence among ITNs, we should operate on all cases. Since this is not possible in clinical practice, we could better understand the cancer risk of ITNs considering several variables as influencing factors on the prevalence of malignancy recorded among the subgroup of operated patients, including the study design (with or without the revision and reclassification of FNAC samples), the overall number of consecutive nodules with available FNAC in a specific period, the prevalence of ITN subcategories among all FNACs, the operation rate, and the final diagnosis at the time of histological assessment. Considering this background, we aimed to properly estimate the prevalence, rate of operation, and risk of malignancy of the indeterminate category of ICCRTC, with the latter being the most reliable system for discriminating low- from high-risk ITNs [11].

## Materials and Methods

### Conduct of Review

This review was conducted according to the Meta-analysis Of Observational Studies in Epidemiology (MOOSE) guidelines [13].

### Search Strategy

A specific strategy to retrieve all original studies citing ICCRTC was planned. Accordingly, the online citation databases Google Scholar and Scopus were searched to find the largest possible number of papers citing ICCRTC. No language restriction was used. A beginning date limit was not used. The last search was performed on February 26, 2022. Additionally, the reference lists of the studies were screened to select additional articles.

### Study Selection

Records found according to the above strategy were fully screened. Original papers reporting data of ITN according to ICCRTC 2014 were included. Articles were not included if (a) they did not cover the field of interest of this systematic review; (b) the histological findings of ITNs was not available; or (c) the data overlapped with other studies. In addition, review articles, editorials, letters, case/series reports (< 10 cases) and pediatric studies were always excluded. Two authors (GF, PT) autonomously reviewed the abstracts of the articles and selected those eligible. In case of disagreement, a consensus was achieved after collegial discussion with the other authors.

### Data Extraction

The following information was extracted independently by two authors (GF, MC) from each study: (1) general study information (authors’ name, year of publication and country origin); (2) modality of enrollment of data of FNACs according to ICCRTC (prospective using ICCRTC during clinical practice or retrospective reclassifying according to ICCRTC of all FNACs performed before 2014); (3) overall number of FNACs performed during the study period; (4) number of ITNs found during the study period; (5) number of ITNs operated on during the study period; (6) number of cancers and benign lesions among ITNs operated on. Separate data extractions were performed for overall ITN, TIR3A and TIR3B. Missing data were obtained from authors of original papers, when appropriate. Data were cross-checked, and a collegial discussion among the authors resolved any discrepancies when present.

### Study Quality Assessment

The risk of bias was independently evaluated by two authors (MC, PT) for each study according to the National Heart, Lung, and Blood Institute Quality Assessment Tool for Observational Studies [14].

### Statistical Analysis

The primary outcomes were (1) the prevalence of cancer among ITNs, TIR3A, and TIR3B; (2) the operation rate among ITNs, TIR3A, and TIR3B; and (3) the prevalence of ITNs, TIR3A and TIR3B among all FNACs. Separate proportion meta-analyses were performed using the DerSimonian and Laird method (random-effect model) [15], where pooled data represent weighted averages according to study sample size. Forest plots displayed the pooled data with 95% confidence intervals (95% CI). The *I*_2_ index was used to evaluate inconsistencies, assessing them as follows: < 25% indicated no heterogeneity, 25–50% indicated mild heterogeneity, 50–75% indicated moderate heterogeneity, and > 75% indicated high heterogeneity. To explore heterogeneity, subgroup analyses and meta-regression analyses were attempted using appropriate covariates (i.e., modality of enrollment of FNAC data according to ICCRTC and sample size). A *p* < 0.05 was regarded as significant. Statistical analyses were performed using OpenMeta[Analyst] (open-source software developed by the Center for Evidence Synthesis in Health, Brown University, Providence, RI, USA).

## Results

### Eligible Articles

**Fig. 1:**
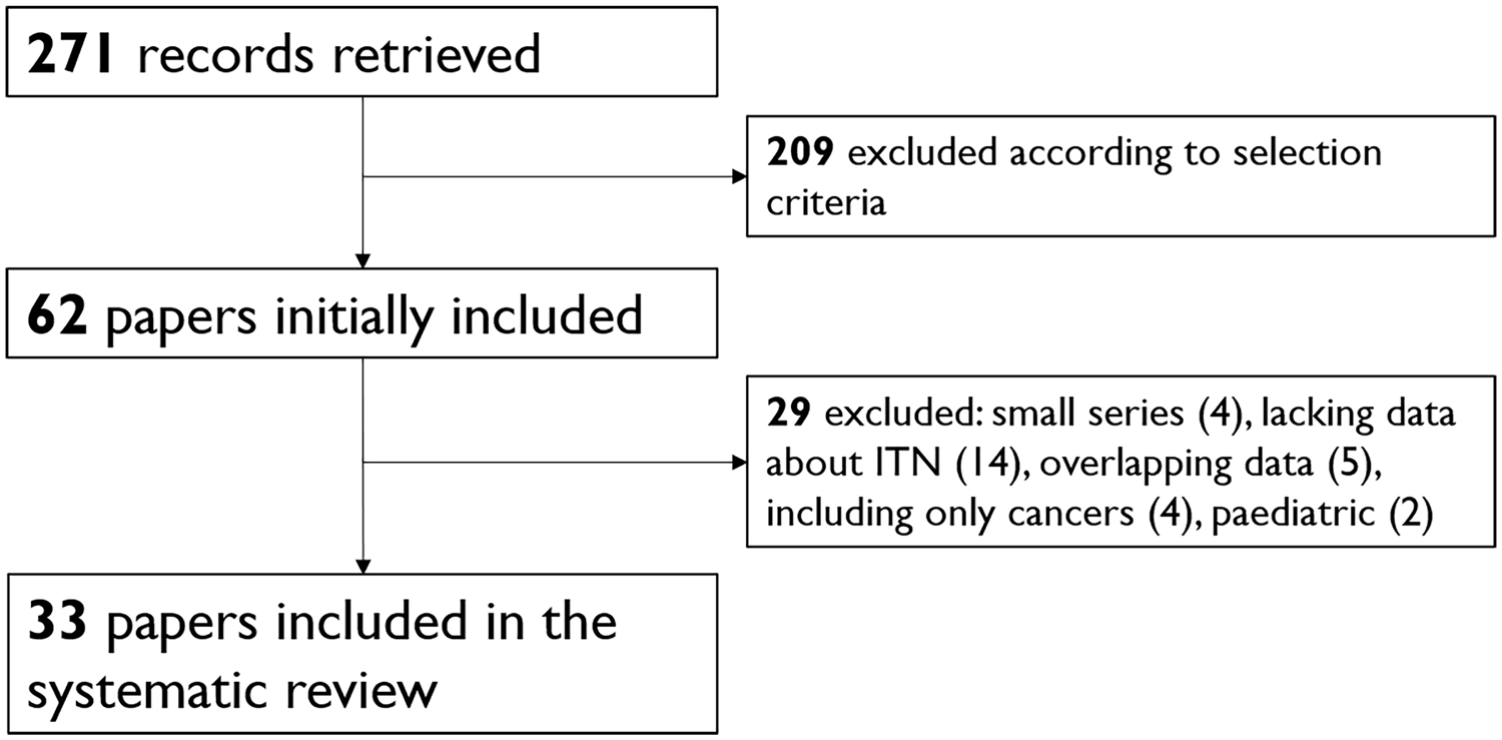
Flow of records found

After excluding duplicates, the online search retrieved 271 articles. According to the above selection criteria, 62 articles were initially selected, and 33 [16–48] were finally included in the systematic review (Fig. 1).

### Qualitative Analysis (Systematic Review)

The 33 articles were published between 2014 and 2021 in scientific journals in the fields of endocrinology (*n* = 23), cytopathology (*n* = 3), medicine (*n* = 3), oncology (*n* = 2), surgery (*n* = 1), and radiology (*n* = 1). The seven oldest studies included nodules originally classified as TIR3 [49], and all cases were reclassified as TIR3A or TIR3B according to the 2014 ICCRTC [7]. Nineteen studies considered nodules first classified as TIR3A or TIR3B, and the remaining 7 papers reported both cases. The overall number of FNACs performed during the study period was available in 20 studies. The total number of ITNs operated on with histological follow-up was 4940, and there were 1516 cases of cancer. Tables 1 and 2 illustrate the main characteristics and full data of the 33 studies.

**Table 1:** Main characteristics and data of the included studies

Ref	First author and year	Journal	Enrolment with respect to 2014 ICCRTC	Country	Overall FNACs	TIR3 recorded	TIR3 among FNAC (%)	TIR3 operated	TIR3 cancers (*n*)	TIR3 cancers (%)	TIR3A operated	TIR3A cancers	TIR3B operated	TIR3B cancers
[16]	Agretti et al. (2014)	J Endocrinol Invest	Before	Italy	153			54	14	25.9	54	14		
[17]	Rossi et al. (2016)	Endocrine	After	Italy	60	27	45.00	27	14	51.9	9	1	18	13
[18]	Grani et al. (2016)	Endocrine	Before	Italy				49	19	38.8	23	6	26	13
[19]	Tartaglia et al. (2016)	J Biol Regul Homeost Agents	Before	Italy	216			52	14	26.9	30	2	22	12
[20]	Trimboli et al. (2016)	Endocrine	Before	Italy				74	19	25.7	41	5	33	14
[21]	Straccia et al. (2017)	Cytopathology	After	Italy	4043	452	11.18	172	41	23.8	63	10	109	31
[22]	Censi et al. (2017)	Frontiers Endocrinol	After	Italy				111	42	37.8	63	14	48	28
[23]	Medas et al. (2017)	Int J Surg	Before	Italy				102	52	51.0	19	4	83	48
[24]	Ulisse et al. (2017)	Int J Endocrinol	Before	Italy				50	15	30.0	23	3	27	12
[25]	Lauria et al. (2018)	Eur J Endocrinology	After	Italy	1169	244	20.87	33	19	57.6	11	4	22	15
[26]	Valabrega et al. (2018)	Front Endocrinol	After	Italy, Switzerland				75	25	33.3	25	1	50	24
[27]	Trimboli et al. (2018)	Endocr Pathol	After	Switzerland	537	63	11.73	51	9	17.6	40	3	11	6
[28]	Rezig et al. (2018)	Metabolomics	After	France, Italy	98	53	54.08	53	18	33.9	46	15	7	3
[29]	Sparano et al. (2018)	J Endocrinol Invest	After	Italy	3264	655	20.07	273	101	37.0	60	15	213	86
[30]	Rullo et al. (2018)	J Endocrinol Invest	Before	Italy				290	84	29.0	128	13	162	71
[31]	Quaglino et al. (2019)	Advances in Medicine	2005–2018	Italy				150	62	41.3	72	15	78	47
[32]	Arena and Benvenga (2019)	Horm Metab Res	2010–2016	Italy	408	184	45.10	184	42	22.8	86	4	98	38
[33]	Fulciniti et al. (2019)	Clin Endocrinol	2013–2018	Italy	115	42	36.52	42	16	38.1	19	2	23	14
[34]	Straccia et al. (2019)	Cytopathology	After	Italy	86	11	12.79	9	8	88.9	2	2	7	6
[35]	Giuliano et al. (2020)	Endocrines	2010–2019	Italy	400	184	46.00	174	51	29.3	86	22	98	29
[36]	Pastoricchio et al. (2020)	Int J Endocrinol	2013–2018	Italy				119	33	27.7	29	5	90	28
[37]	Sponziello et al. (2020)	Endocrine	After	Italy	117	40	34.19	40	9	22.5	9	1	31	8
[38]	Piccardo et al. (2020)	Endocrine	After	Italy, Switzerland				111	27	24.3	67	9	44	18
[39]	Ianni et al. (2020)	NJLM	After	Italy		540		201	49	24.4	78	9	123	40
[40]	Cappelli et al. (2020)	Int J Endocrinol	After	Italy	4321	492	11.39	378	74	19.6	172	17	206	57
[41]	Capezzone et al. (2021)	Front Endocrinol	2009–2019	Italy		188		73	29	39.7	31	3	42	26
[42]	Massa et al. (2021)	J Endocrinol Invest	2011–2018	Italy	1482	302	20.38	134	39	29.1	48	8	86	31
[43]	Pagano et al. (2021)	Endocrine	After	Italy	146	87	59.59	87	34	39.1	5	3	82	31
[44]	Possieri et al. (2021)	Endocrine	After	Italy	34	16	47.06	16	7	43.8	6	3	10	4
[45]	Javalgi and Priyanka (2021)	Thyroid	After	India				33	16	48.5	14	5	19	11
[46]	Celletti et al. (2021)	Radiol Med	After	Italy	128	128		96	28	29.2	17	2	79	26
[47]	Leni et al. (2021)	Cancers	After	Italy	493	117	23.73	39	7	17.9	26	2	12	5
[48]	Poma et al. (2021)	Cancers	After	Italy	9070			1579	499	31.6	1224	343	355	156

Enrollment column indicates how data were collected with respect to 2014 ICCRTC (see the text). Overall FNACs column indicates the number of FNACs performed during the study period. White boxes indicate not available data

**Table 2:** Demographic features and nodule’s size of the included studies

			**Institution**	**Patients**						**Nodule size (mm)**	
				**Male**			**Female**		**Ranges**		**mean ± SD or median**
				***n***	**Age (year)**	***n***		**Age (year)**			
[16]	Agretti et al.	2014	Academic	41	48 ± 8.6	112		45.4 ± 11.7	N/A		N/A
[17]	Rossi et al.	2016	Academic	24/60 overall FNAC	27	36/60 overall FNAC		27	5–50		N/A
[18]	Grani et al.	2016	Academic	13/47	54 ± 12	34/47		54 ± 12	N/A		N/A
[19]	Tartaglia et al.	2016	Academic	79/434 with SIAPEC 2007 and 37/216 with SIAPEC 2014	55	355/434 with SIAPEC 2007 and 179/216 with SIAPEC 2014		55	N/A		N/A
[20]	Trimboli et al.	2016	Referral	13/74 TIR3 operated	48 ± 14.5	61/74 TIR3 operated		48 ± 14.5	4–48		15.4 ± 8.6*
[21]	Straccia et al.	2017	Academic	808/4043 overall FNAC	64	3235/4043 overall FNAC		64	10–140		N/A
[22]	Censi et al.	2017	Academic	44/199	50	155/199		50	N/A		N/A
[23]	Medas et al.	2017	Academic	5/19 TIR3A and 22/83 TIR3B	N/A	14/19 TIR3A and 61/83 TIR3B		N/A	N/A		26.7 ± 14.7 TIR3A 23.4 ± 12.7 TIR3B
[24]	Ulisse et al.	2017	Academic	18/70	58	52/70		58	13–73		58
[25]	Lauria et al.	2018	Academic	199/946	56 ± 13.3	747/946		56 ± 13.3	4–56		14 ± 10
[26]	Valabrega et al.	2018	Academic	25/71	52.3	46/71		52.3	N/A		52.3
[27]	Trimboli et al.	2018	Referral	14/51 TIR3 operated	53 ± 13	37/51 TIR3 operated		53 ± 13	N/A		25.6 ± 13.5 carcinomas
[28]	Rezig et al.	2018	Academic	24	48 ± 14	72		48 ± 14	5–65		21 ± 13
[29]	Sparano et al.	2018	Academic	82/349 TIR3A	N/A	267/349 TIR3A		N/A	N/A		20.6 ± 9.5 of 289 TIR3A not operated 27.0 ± 12.8 of 60 TIR3A operated
[30]	Rullo et al.	2018	Academic	80	52.5	210		52.5	3–65		17.4
[31]	Quaglino et al.	2019	Referral	N/A	52.6	N/A		52.6	17–20		18.5
[32]	Arena and Benvenga	2019	Referral	83 overall FNACs	N/A	325 overall FNACs		N/A	N/A		N/A
[33]	Fulciniti et al.	2019	Academic	37/141	47	104/141		47	≥ 10		N/A
[34]	Straccia et al.	2019	Academic	26/86 overall FNACs	48	60/86 overall FNACs		48	4–32		N/A
[35]	Giuliano et al.	2020	Academic	129/400	46	271/400		46	18.5–26.3		N/A
[36]	Pastoricchio et al.	2020	Academic	N/A	N/A	N/A		N/A	4–60 TIR3A 4–80 TIR3B		23.4 ± 14 TIR3A 29 ± 18 TIR3B
[37]	Sponziello et al.	2020	Academic	N/A	N/A	N/A		N/A	4–64		19.62
[38]	Piccardo et al.	2020	Referral	18/111 TIR3 operated	57.6	93/111 TIR3 operated		57.6	N/A		23
[39]	Ianni et al.	2020	Academic	55/201 TIR3 operated	54.5 ± 14	146/201 TIR3 operated		54.5 ± 14	6–80 TIR3A 4–79 TIR3B		30 TIR3A 20 TIR3B
[40]	Cappelli et al.	2020	Academic	107/378 TIR3 operated	54.7 ± 13.9	271/378 TIR3 operated		54.7 ± 13.9	N/A		19.3 ± 9.2 TIR3 operated
[41]	Capezzone et al.	2021	Academic	42/188 TIR3 recorded 8/73 TIR3 operated	54.5 ± 14.0 TIR3 recorded	146/188 TIR3 recorded 21/73 TIR3 operated		54.5 ± 14.0 TIR3 recorded	7–83 TIR3 recorded		23.9 ± 12.1 TIR3 recorded
[42]	Massa et al.	2021	Academic	90/302 TIR3 recorded	53.5	212/302 TIR3 recorded		53.5	8–80 TIR3A 10–70 TIR3B		24 TIR3A 25 TIR3B
[43]	Pagano et al.	2021	Academic	35/146 overall FNACs	50.5 ± 4.8	111/146 overall FNACs		50.5 ± 14.8	N/A		26.2 ± 16.4
[44]	Possieri et al.	2021	Academic	2/19 suspicious nodules operated	45.5 ± 13.4	17/19 suspicious nodules operated		51.1 ± 15.4	≥ 10		N/A
[45]	Javalgi and Priyanka	2021	Academic	N/A	N/A	N/A		N/A	N/A		N/A
[46]	Celletti et al.	2021	Academic	39/128 TIR3 recorded	54.3	89/128 TIR3 recorded		54.3	> 15		N/A
[47]	Leni et al.	2021	Referral	108/435 patients’ final cohort	59	327/435 patients’ final cohort		59	5–70		18
[48]	Poma et al.	2021	Academic	57/151 PTC elderly 57/206 controls 6/22 elderly FTC and HTC 7/21 controls	> 65 elderly < 65 control	94/151 PTC elderly 149/206 controls 16/22 elderly FTC and HTC 14/21 controls		> 65 elderly < 65 control	N/A		25 ± 16 of 151 PTC elderly 21 ± 12 of 206 controls 47 ± 21 of 22 elderly FTC and HTC 25 ± 9 of 21 controls

In the column reporting the setting of institution “academic” indicates a university while “referral” indicates a public hospital. Data of this table are generally referred to the overall series of cases (patient/nodules) of each study; however, when data are referred to a subgroup, a specification is added. Data with ± refer to mean with standard deviation (SD)

*PTC* papillary thyroid carcinoma, *FTC* follicular thyroid carcinoma, *HTC* Hürthle cell carcinoma, *N/A* not available

### Study Quality Assessment

The assessment of the risk of bias of each study is detailed in the Supplemental data. For all articles, statement of the study question, inclusion and exclusion criteria, exposure of interest (i.e., FNAC), timeframe between exposure and outcome (i.e., histology), and outcome measures were adequate. In two studies, the population was not properly defined [16, 28]. Sample size justification was never reported. Whether the participation rate of eligible persons was at least 50%, it was unclear in 16 studies [16, 17, 19, 24, 26, 28, 30, 33, 35, 37, 41–46]. A loss to follow-up after baseline below 20% was reported in 16 studies [16–20, 22, 23, 27, 30, 31, 34–36, 40, 43, 45].

### Quantitative Analysis (Meta-analysis)

First, the pooled prevalence of cancer among all ITNs was evaluated, and a rate of 32.4% (95% CI 29.2–35.5) was found with high heterogeneity (*I*_2_ 78%). Neither study design (i.e., studies with nodules classified as TIR3A or TIR3B during clinical practice vs. the other ones) nor sample size could explain this finding. However, when the largest study [48] was excluded, an inverse correlation was found between sample size and cancer rate (*p* = 0.025): the higher the sample size was, the lower the cancer rate.

**Fig. 2:**
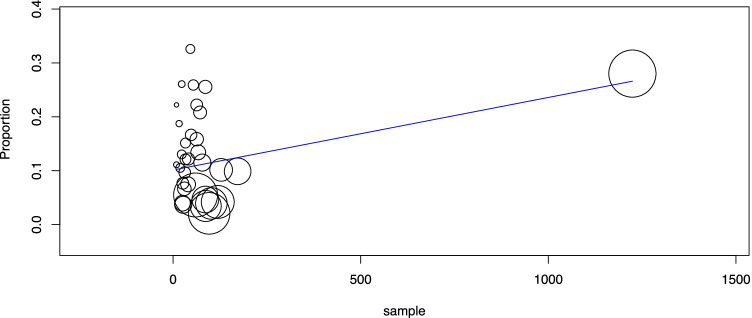
Meta-regression analysis to explore the cancer rate of TIR3A according to study sample size. Any circle identifies one study, and its size differs according to the study weight

Second, the pooled group of 2626 TIR3A cases was analyzed. The cancer prevalence was 12.4% (95% CI 8.8–15.9), with high heterogeneity (*I*_2_ 90%). As described above, heterogeneity was explored according to the study design and sample size. Concerning the former aspect, there was no difference between the subgroup of studies reporting data of nodules reclassified as TIR3A and that of studies including nodules assessed as TIR3A during clinical practice. Regarding the sample size, the meta-regression analysis found a significant linear correlation between sample size and cancer rate (*p* = 0.009): the higher the sample size was, the higher the cancer rate (Fig. 2). However, this result depended on the high weight of the largest study [48], without a significant difference after excluding that series.

**Fig. 3:**
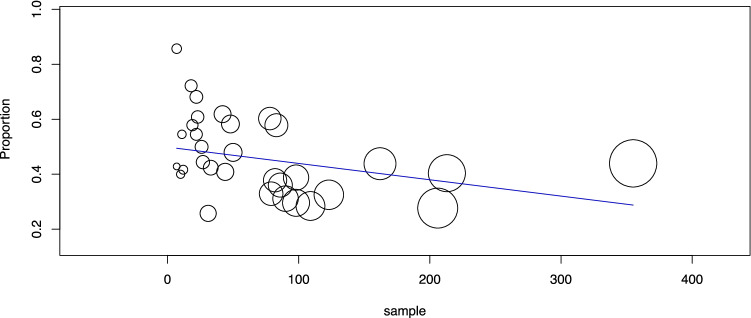
Meta-regression analysis to explore the cancer rate of TIR3B according to study sample size. Any circle identifies one study, and its size differs according to the study weight

Third, the pooled group of 2314 TIR3B nodules was investigated. The cancer prevalence in this category was 44.4% (95% CI 40.1–48.8) with moderate heterogeneity (*I*_2_ 75%). The heterogeneity was explored as described above according to study design and sample size. The study design did not explain the heterogeneity. However, the meta-regression considering the sample size showed a significant inverse correlation between sample size and cancer rate (*p* = 0.031): the higher the sample size was, the lower the cancer rate (Fig. 3). Since the largest study [48] influenced the results of ITN and TIR3A, this was also verified in TIR3B; when excluding that study, the significance of the correlation increased (*p* = 0.001).

**Fig. 4:**
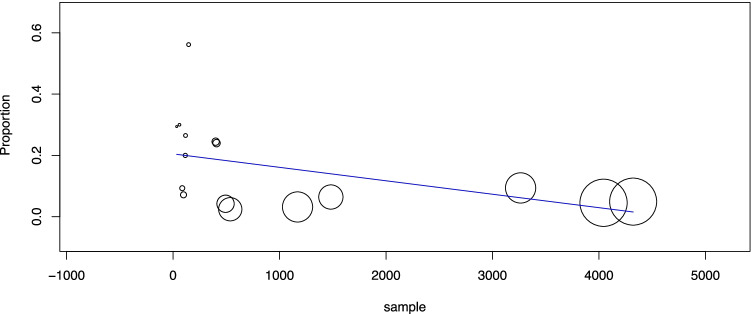
Meta-regression analysis to explore the prevalence of TIR3B among FNACs according to study sample size. Any circle identifies one study, and its size differs according to the study weight

Fourth, the prevalence of ITN, TIR3A, and TIR3B among all FNACs was analyzed. Among those 20 studies reporting the overall number of biopsies performed during the study period, after excluding papers reporting only FNACs with ITN results, there were 16 studies eligible for this analysis [21, 25, 27–29, 32–35, 37, 40, 42–44, 47]. Overall, the prevalence of ITNs among FNACs was 29.6% (95% CI 25–34.1), with high heterogeneity (*I*_2_ 98%). When sample size was used as a covariate, a significant inverse correlation was found between the study sample and ITN prevalence (*p* = 0.002): the higher the sample size was, the lower the ITN prevalence. The pooled prevalence of TIR3A among FNACs was 12.6% (95% CI 10.1–15.2), with high heterogeneity (*I*_2_ 96%), remaining unexplained by meta-regression analysis using sample size as a covariate (*p* = 0.14). The pooled prevalence of TIR3B among FNACs was 12.9% (95% CI 10.5–15.3), with high heterogeneity (*I*_2_ 97%). When sample size was used as a covariate, a significant inverse correlation was observed between sample size and TIR3B prevalence (*p* = 0.04): the higher the sample size was, the lower the prevalence of TIR3B (Fig. 4).

**Fig. 5:**
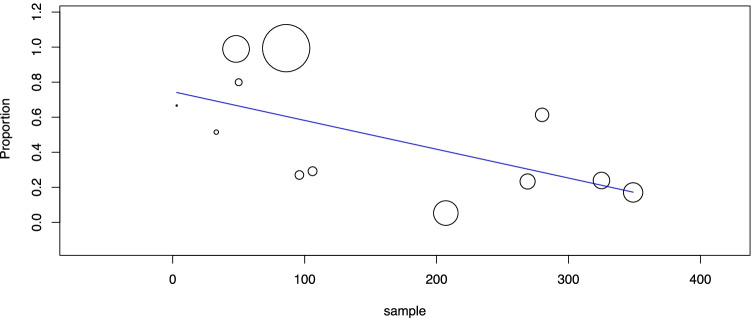
Meta-regression analysis to explore the operation rate among TIR3A cases according to study sample size. Any circle identifies one study, and its size differs according to the study weight

Fifth, the operation rates of ITN, TIR3A, and TIR3B were analyzed. For this analysis, 12 studies were eligible [21, 25, 27, 29, 34, 35, 39–42, 46, 47]. The operation rate of all ITNs was 54.3% (95% CI 38.2–70.5) with high heterogeneity (*I*_2_ 99%), leaving the latter unexplained when performing a meta-regression analysis using sample size as a covariate (*p* = 0.20). When considering the TIR3A group, the operation rate was 48.3% (95% CI 21.9–74.6), with high heterogeneity (*I*_2_ 99%). The latter was explored using the sample size of TIR3A, and a significant inverse correlation was observed between sample size and TIR3A operation rate (*p* = 0.010): the higher the sample size was, the lower the operation rate (Fig. 5). When analyzing the TIR3B group, the operation rate was 75.2% (95% CI 65.9–84.5), with high heterogeneity (*I*_2_ 98%), leaving the latter unexplained when performing a meta-regression analysis using sample size as a covariate.

Finally, the main findings of the present study are summarized in Table 3.

**Table 3:** Summary of findings

	**Overall ITN**	**TIR3A**	**TIR3B**
Cancer prevalence	32.4%	12.4% ^b^	44.4% ^a^
Prevalence among FNACs	29.6% ^a^	12.6%	12.9% ^a^
Operation rate	54.3%	48.3% ^a^	75.2%

^a^The higher the sample size the lower the estimate

^b^The higher the sample size the higher the estimate

^c^This estimate is strongly dependent of one single high-size series

## Discussion

ITN is still a challenge in cytopathology since morphology alone is not able to classify these lesions. Additionally, even if ancillary molecular testing (from single mutational assessment to broader genetic panels) might contribute to more precise and tailored patient management, their use is limited due to their costs. Currently, addressing ITN is still clinically problematic. We can tell our patient that the risk of malignancy is not high, probably mild-to-moderate, even if a cancer cannot be excluded until he is operated upon. The present systematic review aimed to investigate the size of the ITNs. In particular, this study evaluated the prevalence of ITNs among thyroid nodules selected for FNAC, how many patients with ITNs are operated upon, and how many ITNs are malignant once patients are operated upon. Implicitly, these analyses might allow us to better understand the true risk of malignancy of these cases.

First, it should be emphasized that the present systematic review retrieved 271 articles citing ICCRTC, while a previous review [11] found only 95 records. This means that the interest of researchers in ICCRTC is rapidly increasing over time. In addition, while the previous meta-analysis [11] included 1168 ITNs with histological follow-up from 10 studies, we included 4940 cases from 33 studies. This large number of cases should enable us to better illustrate the dimension of ITNs and analyze several aspects. Remarkably, the present systematic review found full data about the flow of ITNs in clinical practice, i.e., their prevalence among FNACs, the resection rate among these patients, and the cancer prevalence among those operated on, and this allowed us to estimate how the cancer rate of ITNs found in histological examination (at the end of the flow) changes according to various covariates. This kind of data could increase the generalizability of the results. In fact, in the field of meta-analyses, the largest the number of covariates available to explore in the pooled results, the more significant the findings. Indeed, the present data form a solid reference for the revised version of ICCRTC. Table 4 compares main data and results of the two studies. It is important to underline that, as a consequence of the larger number of cases, the CIs of the present study were shorter than that of the previous study.

**Table 4:** Comparison between data of the present systematic review and that of a previous one [11]

	**Present systematic review**	**Previous systematic review **[11]
Articles found	271	95
Articles included	33	10
ITN		
Total cases	4940	1168
Cancer prevalence	32.4% (29.2–35.5)	34% (28–41)
TIR3A		
Total cases	2626	441
Cancer prevalence	12.4% (8.8–15.9)	17% (12–22)
TIR3B		
Total cases	2314	727
Cancer prevalence	44.4% (40.1–48.8)	47% (40–55)

Cancer prevalence is reported as pooled result with CI 95%

**Fig. 6:**
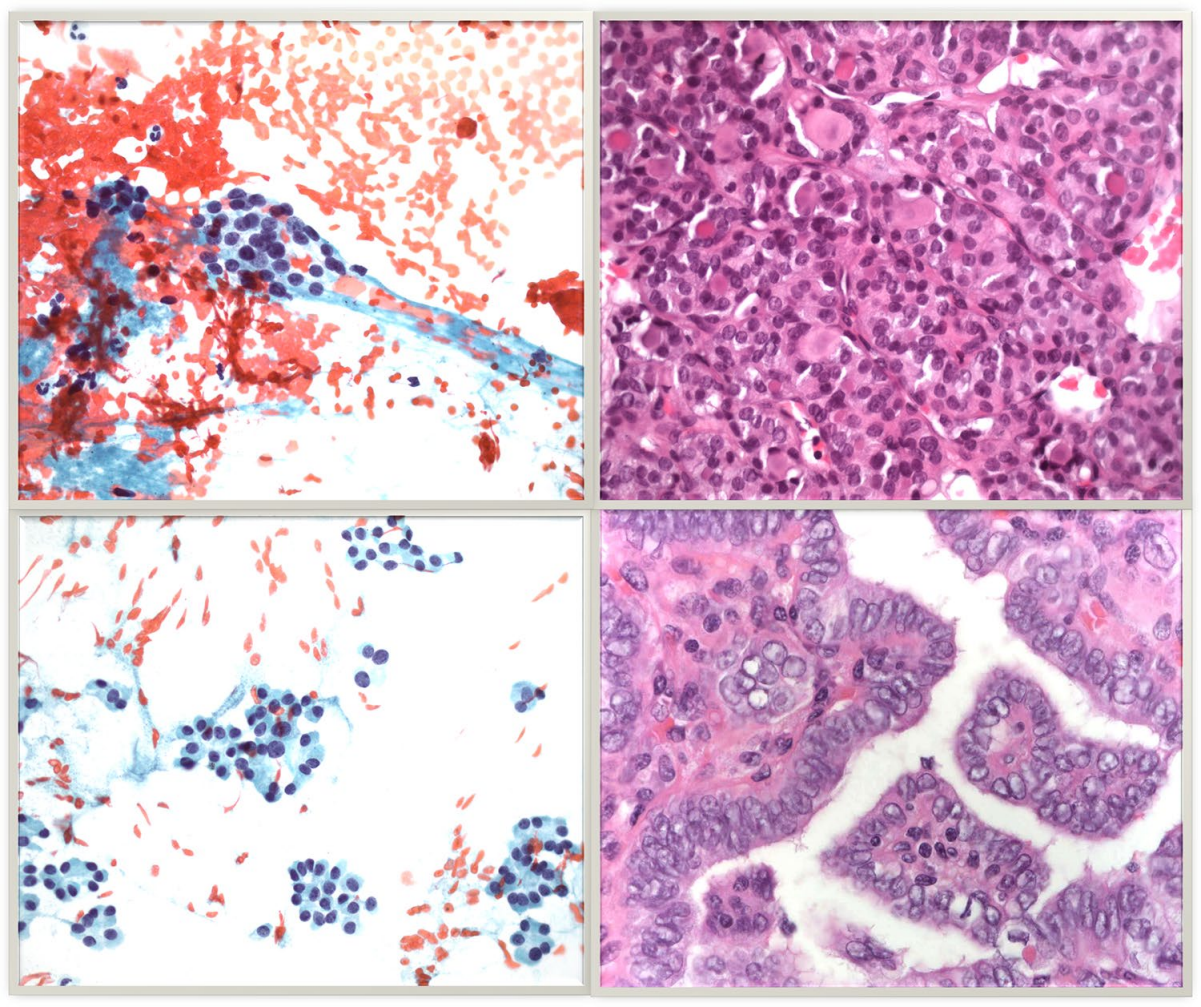
Two cases of thyroid nodules cytologically classified as indeterminate. The upper figures illustrate a nodule classified as TIR3A. Left: Several microfollicular clusters may be observed in a blood-stained background. The cell groups show a certain degree of monotony with slightly enlarged nuclei and finely irregular chromatin. No clear-cut nuclear grooves or intranuclear cytoplasmic inclusions (INCI) are noticed. Right: the postsurgical histological sample showed a follicular variant papillary carcinoma with follicular-patterned lesion where thyrocytes show enlarged nuclei with chromatin clearing and occasional nuclear grooves and INCI. Nuclei also show a tendency to overlap. The follicular lumens contain dense colloid. The lower figures illustrate a TIR3B case. Left: the cytological picture shows abundant cellularity organized into microfollicular structures or trabeculae. Thyrocytes show nucleocytoplasmic atypia with enlarged and pleomorphic nuclei with granular chromatin. The cytoplasm is moderately or well represented, sometimes showing a granular appearance. Colloid is scant. Right: the postsurgical histology showed a classical papillary thyroid carcinoma, made up of papillary clusters of thyrocytes with enlarged nuclei, overlapping and chromatic clearing. Moreover, INCI, nuclear grooves and small nucleoli can be seen

First, while 32.4% of all ITNs were found to be cancerous once patients underwent surgery, a significant difference was found between TIR3A and TIR3B, where the cancer rates were 12.4 and 44.4%, respectively. This finding is of high interest in the current era, in which international terminology harmonization and standardization should be required [50]. In fact, the meta-analyses focused on other FNAC reporting systems did not find a different risk of malignancy between the subclasses of ITN [8–10]. Table 5 summarizes the pooled results obtained in the major meta-analyses about the three major systems of thyroid FNAC. From this point of view, the most relevant difference between ICCRTC and both TBSRTC [6] and UK RCPath [5] is the classification of nuclear atypia. The latter are put into AUS/FLUS of TBSRTC [6] and Thy 3a of UK RCPath [5], which did not aim to separate the subclasses of ITNs according to their risk of malignancy. In contrast, ICCRTC categorized nuclear atypia with potential to be associated with papillary thyroid carcinoma into the “high-risk” category of TIR3B and the other atypia into the “low-risk” TIR3A [7]. Figure 6 illustrates the cytological presentation of two cases of TIR3A and TIR3B with their final histological diagnosis. In this context, it is worth noting a meta-analysis evaluating aspirates with nuclear/cytologic atypia [51] and reporting their significantly higher risk of malignancy. In addition, it has to be mentioned that the risk of malignancy among the subcategories of AUS/FLUS varies significantly, ranging from 15% in “Hürthle cell aspirates with low-risk pattern” to 44% in “Focal cytologic atypia” [52]. Since mild nuclear atypia has been considered in the Bethesda IV class (FN/SFN) of the last TBSRTC version [53], further studies are needed to analyze its impact in clinical practice.

**Table 5:** Cancer rate found in the major meta-analyses about The Bethesda System for Reporting Thyroid Cytology (TBSRTC) and UK Royal College of Pathologists (UK RCPath), and present one about Italian consensus for the classification and reporting of thyroid cytology (ICCRTC)

**Classification system**	**First author of the meta-analysis [ref]**	**Subcategories of ITN**	**Cancer rate (95% CI)**
The Bethesda System for Reporting Thyroid Cytology (BSRTC)	Vuong et al. [9]	AUS/FLUS	31% (24 to 37)
		FN/SFN	29% (26 to 32)
	Straccia et al. [10]	AUS/FLUS	27% (23 to 31)
		FN/SFN	31% (28 to 36)
UK Royal College of Pathologists (UK RCPath)	Poller et al. [8]	Thy3a	25% (20 to 31)
		Thy3f	31% (24 to 39)
Italian consensus for the classification and reporting of thyroid cytology (ICCRTC)	Trimboli [present]	TIR3A	12% (9 to 16)
		TIR3B	44% (40 to 49)

All numbers are rounded to the nearest decimal

*ITN* indeterminate thyroid nodule at FNAC, *95% CI* 95% confidence interval

Second, the most important novel information found in the present systematic review is that there is a strong impact of study sample size on the cancer rate among ITNs, their prevalence among all FNACs and, remarkably, the rate of ITN patients operated upon. Specifically, the prevalence of ITNs, TIR3A and TIR3B among all FNAC was 29.6%, 12.6%, and 12.9%, respectively, while the prevalence of operated nodules was 54.3%, 48.3%, and 75.2%, respectively. Additionally, when we evaluated the impact of sample size on these findings, we observed that the higher the size was, (a) the lower the prevalence of ITNs and TIR3B among FNACs; (b) the lower the operation rate of patients with TIR3A; (c) the lower the cancer rate in TIR3B cases; and (d) the higher the cancer rate in TIR3A. Several variables, such as (a) the different management of any single patient with ITN (and thyroid nodule, of course) in large- and small-volume institutions, (b) the expertise of institutional endocrinologists, pathologists and surgeons, (c) the availability of second-line diagnostic techniques to be used in ITNs (i.e., molecular markers, core biopsy, PET/CT, and other), (d) the rate of patients lost at follow-up, and (e) the preference of patients, could have influenced these findings.

Third, because of these issues, ICCRTC recommendations should be addressed. The suggested actions by ICCRTC are (1) to plan an active clinical observation as the first option in most TIR3A cases with repeated FNAC over time and (2) to operate on patients with TIR3B as the main option. In addition, in the ICCRTC document, since no published data exist regarding both the frequency of ITNs and the risk of malignancy, attempts should be made to keep the TIR3A and TIR3B frequencies < 10%, each with an expected cancer rate < 10% in TIR3A and between 10 and 20% in TIR3B. Based on the data recorded herein, we can affirm that these suggested actions are not fully followed in clinical practice, especially in small-size studies. In fact, more than half of ITNs are addressed via surgery, with a resection rate of 48.3% among TIR3A cases. In addition, the ITN prevalence among FNACs was approximately one-third, with a significant interaction between the TIR3B prevalence and the study sample size.

A comprehensive discussion of these findings is needed. Theoretically, we can expect that a small-size study reports highly selected case series with a potential bias in terms of overestimation of cancer: the smaller the series of ITNs, the more accurate the clinical selection of cases at high risk of cancer (e.g., suspicious US), the higher the operation rate, and the higher the cancer rate at the time of histological examination. From the researchers’ point of view, we have to take into account that, generally, small-sample studies report a positive correlation, which encourages authors (and journal editors) to publish those data. From the clinicians’ standpoint, the creators of guidelines should carefully consider data derived from large-sample studies. As mentioned above, in the 2014 version of ICCRTC, the obvious absence of clinical data on the frequency and cancer rate of TIR3A and TIR3B was underlined. The present findings allow us to obtain solid information about both references. In fact, the frequency of TIR3A and TIR3B was found to be just above 10% among all FNACs, as initially estimated in ICCRTC. However, the cancer rates of TIR3A and TIR3B were quite different from those expected by the ICCRTC board. The results recorded herein can constitute a basis on which to better estimate the frequency of ITNs among FNACs and the risk of malignancy of the two subclasses.

As is typical in systematic reviews, both limitations and strengths of data should be discussed. First, a large number of papers included a retrospective series of ITNs that were reclassified as TIR3A or TIR3B for the study aim. However, data from these studies did not significantly vary from those obtained when pooling studies including nodules classified as TIR3A and TIR3B in clinical practice. Second, those studies with small sample size could have a significant selection bias (in patients with ITNs, in those operated upon, and in those with an initial diagnosis of thyroid nodules). However, this was fully explored and clearly explained in the present study. Third, almost all studies retrieved in the present systematic review were, as largely expected, from Italian authors. Although these results cannot be extended to other countries, they are reliable, as they were derived from institutes that use ICCRTC in their routine clinical practice. Forth, data about non-invasive follicular thyroid neoplasms with papillary-like nuclear features (NIFTP) did not allow any exploration according to operation rate and other covariates. This was due to the fact that NIFTP was not included in the ICCRTC [54]. Then, while the distribution of NIFTP over TBSRTC categories is known [55], its impact on ICCRTC remains unclear.

In conclusion, the present meta-analysis included a very large number of ITNs and corroborates that the cancer rate among ITNs is 32.4%, with a significant difference between low- and high-risk subcategories. Furthermore, this study found that the overall prevalence of ITNs among FNACs was 29.6%, the resection rate of patients with ITNs was 54.3%, and the cancer rate among ITNs was significantly influenced by the study sample size. We advise that the revised version of ICCRTC takes into account these findings as a reference.

## Supplementary Information

Below is the link to the electronic supplementary material.

Supplementary file1 (DOCX 22 KB)
