# Supplementary material for: Thyroid Nodules with Indeterminate FNAC According to the Italian Classification System: Prevalence, Rate of Operation, and Impact on Risk of Malignancy. An Updated Systematic Review and Meta-analysis
Source: Endocr Pathol. 2022 Aug 31;33(4):457–71. doi: 10.1007/s12022-022-09729-x (PMC9712406; doi:10.1007/s12022-022-09729-x)
Supplement: Supplementary file 1 — Supplementary file1 (DOCX 22 KB) [file 12022_2022_9729_MOESM1_ESM.docx]

|  | 1 | 2 | 3 | 4 | 5 | 6 | 7 | 8 | 9 | 10 | 11 | 12 | 13 | 14 | Tot |
| --- | --- | --- | --- | --- | --- | --- | --- | --- | --- | --- | --- | --- | --- | --- | --- |
| Agretti 2014 | Yes | No | Uncl | Yes | No | Yes | Yes | NA | NA | NA | Yes | No | Yes | NA | 6 |
| Arena 2019 | Yes | Yes | Yes | Yes | No | Yes | Yes | NA | NA | NA | Yes | No | Unclear | NA | 7 |
| Capezzone 2021 | Yes | Yes | Uncl | Yes | No | Yes | Yes | NA | NA | NA | Yes | No | No | NA | 6 |
| Cappelli 2020 | Yes | Yes | Yes | Yes | No | Yes | Yes | NA | NA | NA | Yes | No | Yes | NA | 8 |
| Celletti 2021 | Yes | Yes | Uncl | Yes | No | Yes | Yes | NA | NA | NA | Yes | No | No | NA | 6 |
| Censi 2017 | Yes | Yes | Yes | Yes | No | Yes | Yes | NA | NA | NA | Yes | No | Yes | NA | 7 |
| Fulciniti 2019 | Yes | Yes | Uncl | Yes | No | Yes | Yes | NA | NA | NA | Yes | No | No | NA | 6 |
| Giuliano 2020 | Yes | Yes | Uncl | Yes | No | Yes | Yes | NA | NA | NA | Yes | No | Yes | NA | 7 |
| Grani 2016 | Yes | Yes | Yes | Yes | No | Yes | Yes | NA | NA | NA | Yes | No | Yes | NA | 8 |
| Ianni 2020 | Yes | Yes | Yes | Yes | No | Yes | Yes | NA | NA | NA | Yes | No | No | NA | 7 |
| Javalgi 2021 | Yes | Yes | Uncl | Yes | No | Yes | Yes | NA | NA | NA | Yes | No | Yes | NA | 7 |
| Lauria 2018 | Yes | Yes | Yes | Yes | No | Yes | Yes | NA | NA | NA | Yes | No | No | NA | 7 |
| Leni 2021 | Yes | Yes | Yes | Yes | No | Yes | Yes | NA | NA | NA | Yes | No | No | NA | 7 |
| Massa 2021 | Yes | Yes | Uncl | Yes | No | Yes | Yes | NA | NA | NA | Yes | No | No | NA | 6 |
| Medas 2017 | Yes | Yes | Yes | Yes | No | Yes | Yes | NA | NA | NA | Yes | No | Yes | NA | 8 |
| Pagano 2021 | Yes | Yes | Uncl | Yes | No | Yes | Yes | NA | NA | NA | Yes | No | Yes | NA | 7 |
| Pastoricchio 2020 | Yes | Yes | Yes | Yes | No | Yes | Yes | NA | NA | NA | Yes | No | Yes | NA | 8 |
| Piccardo 2020 | Yes | Yes | Yes | Yes | No | Yes | Yes | NA | NA | NA | Yes | No | No | NA | 7 |
| Poma 2021 | Yes | Yes | Yes | Yes | No | Yes | Yes | NA | NA | NA | Yes | No | No | NA | 7 |
| Possieri 2021 | Yes | Yes | Uncl | Yes | No | Yes | Yes | NA | NA | NA | Yes | No | Uncl | NA | 6 |
| Quaglino 2019 | Yes | Yes | Yes | Yes | No | Yes | Yes | NA | NA | NA | Yes | No | Yes | NA | 8 |
| Rezig 2018 | Yes | No | Uncl | Yes | No | Yes | Yes | NA | NA | NA | Yes | No | Uncl | NA | 5 |
| Rossi 2016 | Yes | Yes | Uncl | Yes | No | Yes | Yes | NA | NA | NA | Yes | No | Yes | NA | 7 |
| Rullo 2018 | Yes | Yes | No | Yes | No | Yes | Yes | NA | NA | NA | Yes | No | Yes | NA | 7 |
| Sparano 2018 | Yes | Yes | Yes | Yes | No | Yes | Yes | NA | NA | NA | Yes | No | No | NA | 7 |
| Sponziello 2020 | Yes | Yes | Uncl | Yes | No | Yes | Yes | NA | NA | NA | Yes | No | No | NA | 6 |
| Straccia 2017 | Yes | Yes | Yes | Yes | No | Yes | Yes | NA | NA | NA | Yes | No | No | NA | 7 |
| Straccia 2019 | Yes | Yes | Uncl | Yes | No | Yes | Yes | NA | NA | NA | Yes | No | Yes | NA | 7 |
| Tartaglia 2016 | Yes | Yes | Uncl | Yes | No | Yes | Yes | NA | NA | NA | Yes | No | Yes | NA | 7 |
| Trimboli 2016 | Yes | Yes | Yes | Yes | No | Yes | Yes | NA | NA | NA | Yes | No | Yes | NA | 8 |
| Trimboli 2018 | Yes | Yes | Yes | Yes | No | Yes | Yes | NA | NA | NA | Yes | No | Yes | NA | 8 |
| Ulisse 2017 | Yes | Yes | Uncl | Yes | No | Yes | Yes | NA | NA | NA | Yes | No | No | NA | 6 |
| Valabrega 2018 | Yes | Yes | No | Yes | No | Yes | Yes | NA | NA | NA | Yes | No | No | NA | 6 |

**Supplemental Table 1. Quality assessment**

1. Was the research question or objective in this paper clearly stated?

2. Was the study population clearly specified and defined?

3. Was the participation rate of eligible persons at least 50%?

4. Were all the subjects selected or recruited from the same or similar populations (including the same time period)? Were inclusion and exclusion criteria for being in the study prespecified and applied uniformly to all participants?

5. Was a sample size justification, power description, or variance and effect estimates provided?

6. For the analyses in this paper, were the exposure(s) of interest measured prior to the outcome(s) being measured?

7. Was the timeframe sufficient so that one could reasonably expect to see an association between exposure and outcome if it existed?

8. For exposures that can vary in amount or level, did the study examine different levels of the exposure as related to the outcome (e.g., categories of exposure, or exposure measured as continuous variable)?

9. Were the exposure measures (independent variables) clearly defined, valid, reliable, and implemented consistently across all study participants?

10. Was the exposure(s) assessed more than once over time?

11. Were the outcome measures (dependent variables) clearly defined, valid, reliable, and implemented consistently across all study participants?

12. Were the outcome assessors blinded to the exposure status of participants?

13. Was loss to follow-up after baseline 20% or less?

14. Were key potential confounding variables measured and adjusted statistically for their impact on the relationship between exposure(s) and outcome(s)?
